# Editorial: Pathogenesis in progenitor cells: epigenetics and external influences

**DOI:** 10.3389/fcell.2023.1205372

**Published:** 2023-04-24

**Authors:** Prasad S. Koka, Srinivasa T. Reddy

**Affiliations:** ^1^ Biomedical Research Institute of Southern California, Oceanside, CA, United States; ^2^ Departments of Medicine and Molecular and Medical Pharmacology, David Geffen School of Medicine, University of California, Los Angeles, CA, United States

**Keywords:** progenitor stem cells, differentiation, pathogenesis, stem cell therapy, cell regulation

The pathogenesis of different progenitor stem cells in particular at the precursor stages can have multiple adverse consequences for their differentiation and cell maturation pathways. The onset and development of such multi-lineage cellular pathogenesis are usually triggered by the dysregulation of intrinsic intracellular epigenetic factors. Such consequences can also be caused by the influence of extracellular factors or species that include pathogen invasion, causing abnormal intra- and inter-cellular epigenetic dysregulation mechanisms. We reported and also hypothesized that the susceptibility of hematopoietic stem progenitor cells (HSPC) and endothelial progenitor cells (EPC) to pathogenesis occurs because of HIV and SARS-CoV-2 infection, respectively (Padmanabhan et al., 2020; Koka et al., 2020).

The summarized articles published on this Research Topic comprise the following relevant mechanisms of cellular pathogenesis (Koka and Reddy). Impairment of the cellular levels and functions due to abnormal molecular events within the cells is a topic of high importance as summarized in the following articles. The cited articles on the Research Topic also evaluate potential stem cell therapies to prevent or minimize the deleterious consequences that cause pathogenesis.

Fetal and adult HSC/HSPCs are susceptible to both benign and malignant hematopoiesis but the latter in particular involves mechanisms and interactions of different intra- and extra-cellular factors (Jassinskaja and Hansson). Single-cell proteomics using the recent advances and applications of mass spectrometry (MS)-based investigations has been proposed to analyze the cellular proteomes to study the intra- and inter-molecular interactions of these cells that drive the leukemic pathogenesis. The potential roles of extracellular transcription factors including stem cell growth factors are also discussed.

The transcriptional repressor GFI1 is shown to regulate the differentiation of hematopoietic stem progenitor cells (HSC/HSPC) into myeloid and lymphoid lineages in a dose-dependent manner of the expression levels of the two alleles in this transcription factor and a transplanted murine model (Xie et al.). Gfi1-KO and GFI1-KD HSCs retain their functions toward engraftment and self-renewal but lose their ability to differentiate into myeloid and lymphoid cells.

The roles of RNA modification mechanisms resulting in N6-methyladenosine (m^6^A) in different progenitor stem cell types have been discussed for useful applications in stem cell transplantation (SCT) to improve the beneficial results of regenerative medicine (Wei et al.). These pluripotent stem cells include embryonic stem cells (ESCs), induced pluripotent stem cells (iPSCs), and adult stem cells. The suggested or proposed goal of the targeted subject in the cited article is to increase self-renewal and differentiation in a preferred manner using SCT technology.

The potential role of the recently discovered innate lymphoid cells (ILCs) in the development of malignancy and their involvement in immunotherapy has been proposed and discussed (Sugimura and Wang). The ILCs are differentiated from the common lymphoid progenitors (CLPs) to play beneficial and deleterious roles. Events such as the pro-tumor TGF-beta mediated trans-differentiation of ILC3 into ILCreg may be delved into in the context of ILC-directed immunotherapy of malignancies such as colorectal cancer (CRC).

Normal fetal neural stem cell (NSC) development is impaired due to the intrauterine growth restriction (IUGR) shown in a murine model but resumed subsequently following maternally induced hypoxia (Chou et al.). IUGR purportedly delayed cell cycle progression in the G2/M phase during intra-cellular nuclear migration, but the relieved NSCs due to the onset of maternal hypoxia re-established the otherwise aborted fetal cortical neurogenesis and yielded the production of layer-specific neurons in this process.

Osteonecrosis of the femoral head (ONFH) is a condition of the hip for which cell therapy-based core decompression (CD) has been suggested rather than total hip arthroplasty (THA), especially for secondary hip arthritis or advanced stage femoral head collapse (Wang et al.). This is an example of CD aided by bone marrow transplantation or cell therapy that can overcome the seemingly less efficacious bone grafting (BG) alone to treat this condition that occurs from THA.

A comparison of the therapeutic applications of the tissue-isolated multipotent mesenchymal stem cells (MSC) and laboratory-generated embryonic stem cell (ESC)-like induced pluripotent stem cells (iPSC) Thanaskody et al. has been presented. The advantages and disadvantages of MSCs versus iPSCs for applications and their usefulness for stem cell therapies have been discussed. In this context, allogeneic MSCs are also amenable and can waive selective patients’ autologous cell requirements for advancing beneficial stem cell therapies.

Thus the collection of articles published in this Research Topic has made forays into different types of pathogenesis of multiple origin precursor progenitor stem cells, when such abnormal events occur during the course of a “disease” state and induced by various natural or species-cell-molecule ecosystem phenomena.
